# Seasonal Growth of *Zygophyllum dumosum* Boiss.: Summer Dormancy Is Associated with Loss of the Permissive Epigenetic Marker Dimethyl H3K4 and Extensive Reduction in Proteins Involved in Basic Cell Functions

**DOI:** 10.3390/plants7030059

**Published:** 2018-07-15

**Authors:** Janardan Khadka, Narendra S. Yadav, Gila Granot, Gideon Grafi

**Affiliations:** French Associates Institute for Agriculture and Biotechnology of Drylands, Jacob Blaustein Institutes for Desert Research, Ben-Gurion University of the Negev, Midreshet Ben-Gurion 84990, Israel; janak_khd@hotmail.com (J.K.); nsyadava2004@gmail.com (N.S.Y.); granotg@exchange.bgu.ac.il (G.G.)

**Keywords:** epigenetics, H3K9 methylation, H3K4 methylation, DNA methylation, seasonal climate change, summer dormancy, heat shock proteins, ribosomal proteins, *Zygophyllum dumosum* Boiss

## Abstract

Plants thriving in desert environments are suitable for studying mechanisms for plant survival under extreme seasonal climate variation. We studied epigenetic mechanisms underlying seasonal growth cycles in the desert plant *Zygophyllum dumosum* Boiss., which was previously shown to be deficient in repressive markers of di-methyl and tri-methyl H3K9 and their association with factors regulating basic cell functions. We showed a contingent association between rainfall and seasonal growth and the epigenetic marker of dimethyl H3K4, which disappears upon entry into the dry season and the acquisition of a dormant state. DNA methylation is not affected by a lack of H3K9 di-methyl and tri-methyl. Changes in methylation can occur between the wet and dry season. Proteome analysis of acid soluble fractions revealed an extensive reduction in ribosomal proteins and in proteins involved in chloroplasts and mitochondrial activities during the dry seasons concomitantly with up-regulation of molecular chaperone HSPs. Our results highlight mechanisms underlying *Z. dumosum* adaptation to seasonal climate variation. Particularly, summer dormancy is associated with a loss of the permissive epigenetic marker dimethyl H3K4, which might facilitate genome compaction concomitantly with a significant reduction in proteins involved in basic cell functions. HSP chaperones might safeguard the integrity of cell components.

## 1. Introduction

In nearly all agricultural regions, abiotic stresses such as drought, salinity, and temperature extremes reduce average yields for most major crop plants by more than 50%, which presents a huge impediment to feeding an ever-growing world population [1]. With the expected changes in the global climate, environmental stresses are likely to increase in severity. This emphasizes the need for better understanding of the biological basis for abiotic stress tolerance in plants. Conceivably, plants flourishing in harsh desert environments might possess novel mechanisms for stress tolerance and are most suited for studying biochemical and molecular mechanisms for plant survival under variable seasonal climate conditions. The desert plant *Zygophyllum dumosum* Boiss. (bushy bean caper), which is a Saharo-Arabian phytogeographical element, inhabits desert regions in Israel (Judea desert and the central Negev) and Egypt (central Sinai) [2]. It is adapted to a variable, desert environment through multiple morphological and molecular mechanisms that act together to bring about the survival of the plant to a combination of stresses prevailing in the desert ecosystem. On entry into the summer, *Z. dumosum* shed its leaflets leaving the thick, wax-covered petioles alive and capable of survival for two full growing seasons [3]. During the summer, *Z. dumosum* in the field enters true dormancy, which is characterized by cessation of cambial activity and by the failure to reactivate this activity and resume growth even after an ample supply of water [4]. Dormancy in the remaining petioles is facilitated by a significant reduction in nuclear size/volume resulting from genome compaction [3]. Therefore, summer dormancy likely provides a major strategy for ‘drought survival’ during the dry season [5,6]. Previous studies showed that *Z. dumosum* as well as other *Zygophyllaceae* species, which usually inhabit dry and semidry regions worldwide, do not possess the repressive epigenetic markers of di-methyl and tri-methyl lysine 9 of histone H3 (H3K9) but do contain mono-methyl H3K9 [3,7]. Histone methylation is specific and is catalyzed by various enzymes that add a methyl group to a specific lysine or arginine residue. For example, SET domain-containing histone methyltransferases such as KRYPTONITE/SUVH4, SUVH5, and SUVH6 in A. thaliana are enzymes that specifically methylate histone H3 at lysine 9 [8], which generates a binding site for CHROMO-containing proteins such as CHROMOMETHYLASE3 (CMT3) [9]. This is an enzyme that maintains cytosine methylation especially in the context of CHG (where H is C, A or T) [10,11]. Consequently, methylated CHG sites serve as binding sites for SET and RING finger Associated (SRA)/YDG domains-containing proteins such as KRYPTONITE/SUVH4. This generates a feedback loop that expands both DNA and H3K9 methylation, which leads to chromatin compaction and gene silencing [12]. Even though multiple mechanisms (e.g., morphological, physiological, and molecular) were evolved in plants to survive their ever-changing environment, epigenetic means appear to be central in controlling gene expression and are, therefore, important for stress memory and adaptation in plants [13,14]. Plant response to and recovery from stresses are complex and involve the activation/repression of hundreds of genes responsible for deploying a variety of defense mechanisms to enable the plant to survive [15]. Most research related to stress tolerance has been performed under controlled growth conditions using model plants such as *Arabidopsis thaliana* whose genome has been sequenced and for whom a vast array of molecular tools has been developed [16]. This approach has been proven successful in isolating genes whose manipulation in plants (i.e., transgenic plants) has often conferred stress tolerance under growth-room conditions [17,18,19,20] but had only marginal effect or needs further examination under field growth conditions [20,21]. This is probably because, under field-growth conditions, plants are often subjected to various combinations of stresses that induce a unique response, which is different from the sum of responses to each stress when given separately [22]. Therefore, studying plants in their natural habitats (e.g., desert plants) might be a reasonable approach for unraveling novel mechanisms controlling tolerance to drought in combination with other stresses prevailing in the desert ecosystem (e.g., high temperature, high irradiation, and salinity). In this study, we investigated how the seasonal growth cycle (transition from growth to dormancy to growth) of the desert plant *Z. dumosum* in its natural habitat (characterized by extreme seasonal climate variation) is associated with epigenetic modifications and with the expression of factors regulating basic cell functions (e.g., protein synthesis, photosynthesis).

## 2. Materials and Methods

### 2.1. Field Site

The study was conducted at the Sede Boqer research area on a southeast-facing rocky slope (30°51′ N 34°46′ E; elevation 498 m), which is dominated by *Zygophyllum dumosum* Boiss. Specific features of the area have been described elsewhere [23,24]. The study spanned the years of 2007, 2009, and 2010 with an average rainfall of 164 mm (winter of 2006–2007), 42 mm (winter of 2008–2009), and 155 mm (winter 2009–2010), respectively.

### 2.2. Plant Materials, Acid Extraction of Proteins, and Immunoblotting

Petioles were collected from *Z. dumosum* shrubs located on the southeast facing slope at all elevations (random sampling) every month and kept at −80°C until used. Notably, old petioles were used in all experiments presented in this study. Petioles were extracted with 2% trichloroacetic acid (TCA) in NETN buffer (100 mM NaCl, 1 mM EDTA, 20 mM Tris, pH 8, and 0.5% NP-40) and supplemented with protease inhibitor cocktail (Sigma, St. Louis, MO, USA) as described [3]. Protein concentration was determined by the Bradford reagent (BioRad, Hercules, CA, USA). Acid-soluble proteins (10 μg) enriched with histones were resolved by 17% SDS/PAGE and immunoblotted with anti-dimethylated H3K4 (Cell Signaling Technology, Danvers, MA, USA). Immuno-detection was performed using a secondary antibody of goat anti-rabbit alkaline phosphatase conjugate (Sigma) and BCIP/NBT substrate (Roche, Basel, Switzerland). Analysis of HSP proteins was performed by immunoblotting using anti-HSP70 (AS08 371, Agrisera AB, Vannas, Sweden) and anti-HSP17.6 (AS07 254, Agrisera AB, Vannas, Sweden). 

### 2.3. Acid Soluble Protein Extraction for Proteome Analysis

Acid soluble proteins were extracted with 2% trichloroacetic acid and re-suspended with NETN, which was described in Reference [3]. For proteome analysis, further purification of acid soluble proteins was completed by using the methanol-chloroform method. A total of 0.8 mL of methanol were added to 0.2 mL of acid soluble proteins and mixed well by vortexing. Chloroform (0.2 mL) was added and the samples were vortexed and centrifuged (10 s at 10,000× *g*). Lastly, precipitated proteins were washed by adding 0.6 mL methanol and the samples were mixed and centrifuged (2 min at 10,000× *g*) to pellet the protein. The protein pellet was dried and re-suspended in phosphate buffered saline (PBS). Protein concentration was measured by using the Bradford assay (BioRad, Hercules, CA, USA).

### 2.4. Proteome Analysis

Acid soluble proteins extracted from petioles collected in the field in April and October 2007 were subjected to proteome analysis by the proteomic services of The Smoler Protein Research Center in Technion, Israel. Proteins were digested with trypsin followed by separation and mass measurement on LC-MS/MS on LTQ-Orbitrap. Mass spectrometry proteomics profiling and initial processing of the results was done by using Discoverer 1.4 against the Arabidopsis Uniprot database. All the identified peptides were filtered with high confidence, top rank, mass accuracy, and a minimum of two peptides. High confidence peptides passed the 1% FDR threshold (FDR = false discovery rate, which is the estimated fraction of false positives in a list of peptides). The area of the protein was calculated from the average of the two to three most intense peptides from each protein. Protein names and gene ontology (GO) annotations were retrieved from UniProt. GO categorization was completed by using PANTHER classification system software [25].

### 2.5. DNA Extraction and Methylation Analysis

For DNA methylation analysis, genomic DNA was extracted from petioles as described [26]. DNA was further treated with RNase A to remove RNAs followed by chloroform-isoamyl alcohol extraction and ethanol precipitation. DNA was quantified by measuring absorbance at 260 nm using nanodrop ND-1000 spectrophotometer and the quality of DNA was checked by using ethidium-bromide (EtBr) staining after running on 1.2% agarose gel.

DNA methylation analysis was done by using the MS-RAPD-PCR method [27]. Restriction digestion of 1 μg DNA was performed separately with methylation-sensitive restriction enzymes *Msp*I and *Hpa*II. Then digested DNA as well as undigested controls were subjected to PCR using primers indicated at the bottom of each image. For analysis of 18S rDNA, we used 18S-F-CTAGAGCTAATACGTGCAACAAACC and 18S-R-GATTGTACTCATTCCAATTACCAGAC. The PCR amplifications were performed in 10 μL of a reaction mixture containing 5 μL of 2× Taq PCR MasterMix (TIANGEN, Beijing, China), 500 nM primers, and a 20 ng template DNA. The amplification was performed in a Bio-rad T100 thermal cycler using the following program: 95 °C for 4 min, 20–40 cycles of 95 °C, 30 s, 34 °C or 55 °C, and 30 s, 72 °C, 2 min, which was followed by a final extension of 72 °C, 5 min. The PCR products were resolved on a 1.5% agarose gel and visualized by EtBr staining.

## 3. Results

### 3.1. Histone H3K4 Methylation is Associated with Seasonal Growth of Z. dumosum

**Figure 1:**
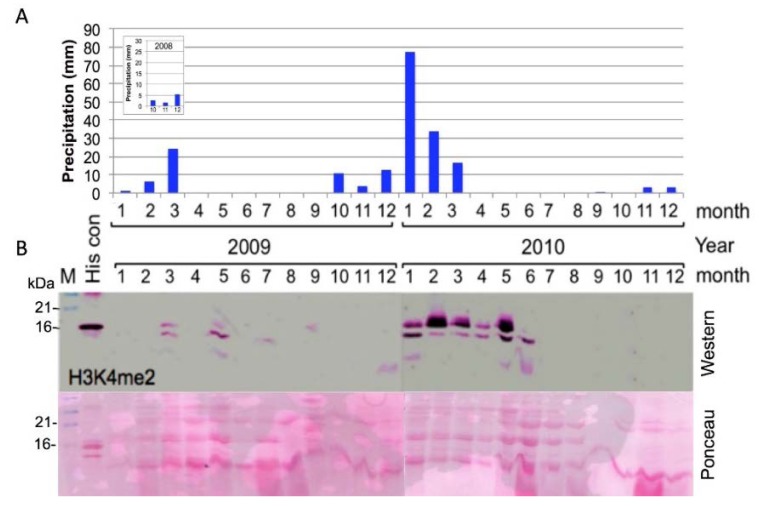
A positive correlation between rainfall (precipitation) and the level of H3K4me2 in *Z. dumosum* petioles during the years 2009 and 2010. (**A**) The cumulated rainfall in each month of 2009 and 2010. Inset displays the rainfall amount in October, November, and December of 2008. (**B**) Immunoblotting analysis of the level of histone H3 di-methylated at lysine 4 (H3K4me2) in *Z. dumosum* petioles during the years of 2009 and 2010. Acid soluble proteins (5 μg) were separated on 15% SDS/PAGE and blotted onto nitrocellulose membrane, which was stained with ponceau (**lower** panel) and was immunoblotted with anti-H3K4me2 (**upper** panel). Note the high level and the extended presence of H3K4me2 in petioles collected during the rainfall-rich year of 2010.

We examined how rainfall levels affect *Z. dumosum* seasonal growth and gene expression activity by monitoring the level of histone H3K4 dimethyl (H3K4me2), which is a histone modification associated with transcriptionally active chromatin. Histones prepared from petioles collected each month during the years 2009 and 2010 were subjected to immunoblotting using specific H3K4me2 antibodies. The results were superimposed on the precipitation data during 2009 and 2010, which showed a clear correlation between rainfall amounts during the winter months and H3K4me2 in *Z. dumosum* petioles (Figure 1). Accordingly, H3K4me2 could not be detected in petioles collected in January and February of 2009, which probably resulted from insufficient and variable distribution of rainfall during the winter of 2008–2009 (42 mm, Figure 1A data from https://ims.data.gov.il/). This rendered one-year-old petioles incapable of resumption of gene expression activity and consequently growth was halted. However, two events of rainfall at the beginning and the end of March (4.3 and 18.8 mm, respectively) allowed for growth to resume, which was accompanied by the occurrence of H3K4me2 at low levels, which persisted up to May. However, during the winter of 2010, which was rich in precipitation (155 mm) and displayed a well-balanced rainfall distribution (https://ims.data.gov.il/), high levels of H3K4me2 were recovered from petioles collected during January and persisted up to June, which demonstrate an extended growth period. H3K4me2 is completely erased thereafter and could not be detected in petioles from July to December of 2010. This might lead to cessation of transcriptional activity, which is consistent with the reduction in nuclear size and acquisition of compact chromatin observed in *Zygophyllum* petioles during the dry season [3].

### 3.2. DNA Methylation in Z. dumosum

**Figure 2:**
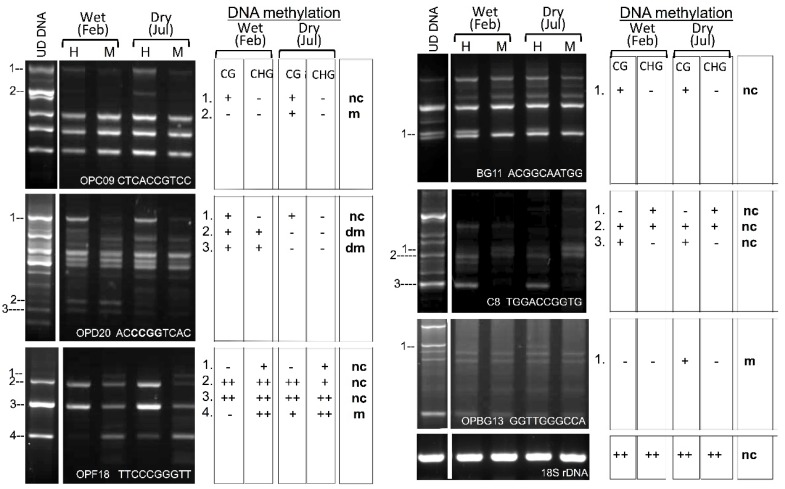
DNA methylation in *Z. dumosum* petioles collected during the wet (February) and the dry (July) seasons. A methylation-sensitive random amplified polymorphic DNA-polymerase chain reaction assay was performed using *Hpa*II (H) and *Msp*I (M) methylation sensitive enzymes and random primers (primer sequence is given at the bottom of each image). Polymorphic fragments are marked by numbers and the DNA methylation contexts (CG or CHG) is interpreted in the table shown on the right where ‘+’ indicates positive methylation and ‘−’ no methylation. Note that 18S rDNA are highly methylated at both CG and CHG contexts and no change (nc) in methylation is observed between the wet and the dry season. m and dm indicate that the restriction site is undergoing methylation or demethylation, respectively, when transitioning into the dry season.

Because DNA methylation is coupled with histone methylation, we examined how the absence of di-methyl and tri-methyl H3K9 affects DNA methylation especially in the CHG context. Since genomic data of *Z. dumosum* does not exist, to address this question, we employed methylation-sensitive random sampling amplified with a polymorphic DNA-polymerase chain reaction (MS-RAPD-PCR, [27]. Genomic DNA isolated from *Z. dumosum* petioles collected during the wet (February 2010) and the dry (July 2010) seasons were subjected to digestion by the methylation-sensitive enzymes *Hpa*II and *Msp*I, which recognize the CCGG site but differ in sensitivity to methylation. Accordingly, *Hpa*II is sensitive when either cytosine is methylated while *Msp*I is sensitive only when the external cytosine is methylated. This distinguishes between CG and CHG methylation. Using multiple random primers as well as specific primers for the 18S ribosomal DNA, we found that CHG methylation does occur in the genome of *Z. dumosum* when multiple polymorphic fragments were recovered by PCR from *Msp*I digest (Figure 2). Accordingly, out of 15 polymorphic fragments examined, 6 fragments were exclusively methylated at the CG context, 2 at the CHG context, and 7 fragments at both CG and CHG contexts. Furthermore, 10 of these fragments showed no change in methylation in the winter and the summer, 3 underwent methylation, and 2 fragments underwent demethylation, which demonstrated that changes in methylation do occur when transitioning into the summer. Therefore, DNA methylation at the CHG context persists in *Z. dumosum* in spite of the lack of H3K9 di-methyl and tri-methyl. Entry into the summer is accompanied by reprogramming DNA methylation.

### 3.3. Seasonal Changes in Heat Shock Proteins (HSPs)

**Figure 3:**
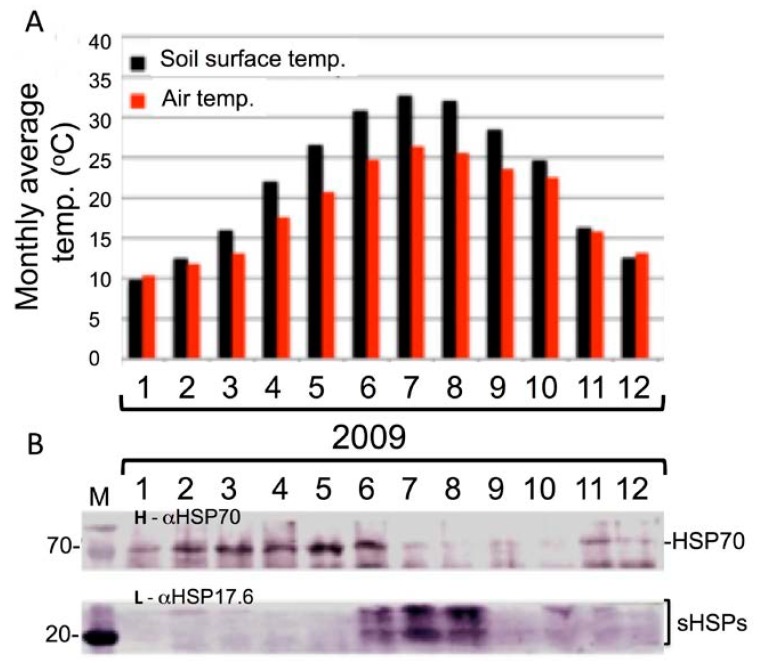
Expression of heat shock proteins in *Z. dumosum* petioles during the year of 2009. (**A**) The monthly average temperature (°C) of the air (red column) and the soil surface (black column) in each month of 2009. (**B**) Total proteins extracted from petioles collected in each month of 2009 were separated on 12% SDS/PAGE, and blotted. The membrane was stained with ponceau and cut into two parts containing high (H) and low (L) molecular weight proteins. The high and low membranes were subjected to immunoblotting using anti-HSP70 (αHSP70) and anti-HSP17.6. (αHSP17.6), respectively. Note that antibodies to HSP17.6 can potentially recognize other small HSPs (sHSPs) since they share significant amino acid sequence homology. The molecular mass (M) is given on the left in kDa.

Petioles persist during the dry, hot season and resume activity in the following wet season. We assumed that, during the dry season, HSPs commonly known as molecular chaperones might be involved in maintaining the integrity of petiole’s cells by avoiding mis-folding and aggregation of proteins. We, therefore, analyzed *Z. dumosum* response to seasonal temperature variation by measuring the level of HSPs in petioles in each month of the year of 2009. We used immunoblotting with antibodies to HSP70 and to small HSP17.6 proteins. Small HSPs are known to be induced following exposure of plants to various stress conditions and are assumed to play a role in stress tolerance [28]. The results show (Figure 3B) a distinct expression pattern of HSP70 and HSP17.6 in *Z. dumosum* petioles during the year. While HSP70 is expressed in petioles collected during winter and spring months, its expression was reduced significantly during the summer months (July to October). However, HSP17.6 was restrictively expressed during the months displaying the highest temperatures, which are the months of June to August. No expression or low expression is evident in other months of the year.

### 3.4. Changes in Acid Soluble Proteins in Petioles During the Transition from the Wet to the Dry Season 

**Figure 4:**
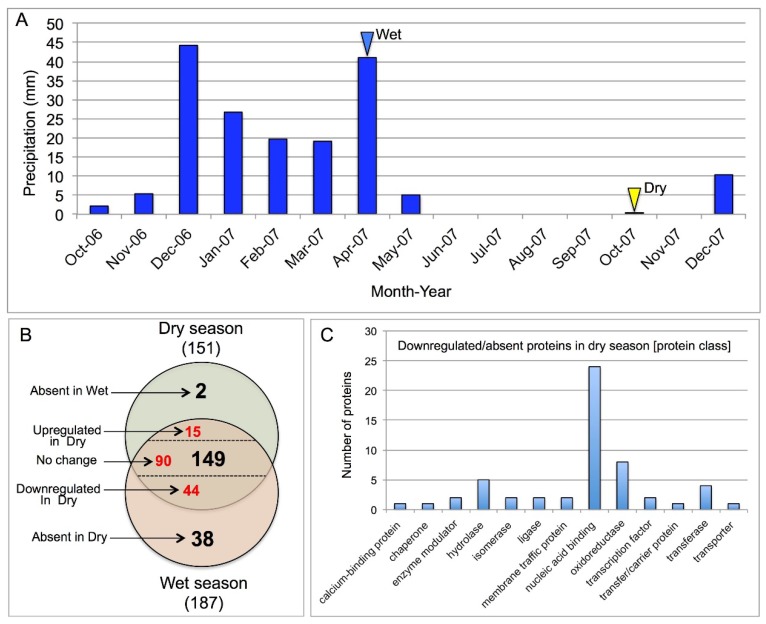
Proteome analysis of acid soluble proteins extracted from *Z. dumosum* petioles in the wet and the dry seasons. (**A**) The cumulated rainfall in the indicated months of 2006 and 2007. Blue and yellow arrowheads indicate the wet and the dry months, respectively, in which petioles were collected for proteome analysis. (**B**) The venn diagram shows the number of proteins recovered from petioles during the wet and the dry seasons. (**C**) Categorization analysis (Protein class) using the PANTHER classification system of 82 proteins that were down-regulated or absent in the dry season petioles. Notably, most proteins related to the nucleic acid binding class are ribosomal proteins (see Table 1).

The dormant state assumed by *Z. dumosum* during the summer is retained even when *Zygophyllum* shrubs were irrigated when they failed to evoke cambial activity or leaf development [4]. Obviously, at the onset of entry into a dormant state, at the beginning of the summer, *Z. dumosum* undergoes extensive changes in the gene expression pattern associated with chromatin compaction [3] and disappearance of H3K4me2, which leads to a significant reduction in cellular activities. To address how the reduction in cell activity is achieved, we performed proteome analysis to identify basic proteins including histones and ribosomal proteins operating during the wet and the dry seasons. To this end, petioles were collected from *Z. dumosum* in 2007 during the wet season (April 2007) and the end of the dry season (October 2007) (see Figure 4A). These petioles were subjected to acid extraction (to enrich for basic proteins) followed by proteome analysis by LC-MS/MS and identification by Discoverer software against the Arabidopsis Uniprot database. Notably, the analysis of the permissive epigenetic marker dimethyl H3K4 during the winter of 2007 was previously described [3], which revealed persistence of dimethyl H3K4 up to July. This further supported the correlation between rainfall amount and distribution as well as growth and the persistence of H3K4me2 in *Z. dumosum* petioles. The proteome data (Figure 4B and Supplementary Table S1) revealed 189 proteins in which 187 were recovered from winter petioles and 151 from summer petioles. Among the 187 proteins recovered in winter petioles, 82 proteins were either significantly reduced (44 proteins) or were absent (38 proteins) in summer petioles (Figure 4B, Supplementary Table S1). Functional categorization (PANTHER classification system) of these 82 proteins revealed (Figure 4C) that among the 55 proteins recognized in the protein class category, 24 proteins are involved in nucleic acid binding (most of which are ribosomal proteins; Table 1), five proteins are chloroplastic hydrolases (Table 1), and eight proteins are essentially mitochondrial oxireductases/dehydrogenases (Table 1). Among the 151 proteins recovered in summer petioles, 17 proteins were either upregulated (15 proteins) or were absent in winter petioles and are induced in the summer (two proteins) (Table 2). Among these 17 upregulated proteins, three proteins are related to the ubiquitin system including the polyubiquitin precursor protein (related to Arabidopsis UBQ10, At4g05320) and ubiquitin conjugates known as the Ubiquitin-60S ribosomal protein L40-2 and the Ubiquitin-40S ribosomal protein S27a-2. Notably, the levels of core histone proteins remain essentially unchanged in the wet and the dry season except for a notable reduction in histone H4 during the dry season (Supplementary Table S1).

We also identified a small heat shock protein called HSP17.4 whose level is significantly increased (more than six-fold) in petioles during the dry season when compared to the wet season. In addition, upregulation in *Z. dumosum* petioles during the dry season include two HSP90 proteins, which are related to the *Arabidopsis* HSP90 cluster III having 2–3 introns [29] as well as HSP70B (related to Arabidopsis At1g16030), which is implicated in response to heat stress [30].

## 4. Discussion

Plant species flourishing in desert environments have evolved multiple mechanisms that enable them to respond to extremely variable seasonal climate conditions occurring during the wet and the dry seasons. Obviously, the dry summer poses challenges to desert plants including long periods of high temperatures, high radiation, and water scarcity. Yet, the summer is stable and predictable and many plants thriving in arid and semi-arid regions shut down growth and enter into a dormant state, which is an adaptive trait conferring survival under severe drought conditions [31,32]. Most studies related to summer dormancy focused on perennial grasses inhabiting arid and semi-arid regions, which showed that dormancy is commonly induced by several environmental factors including long days, high temperatures, and a water deficit [33,34,35]. In *Z. dumosum*, a water deficit appears to be the major factor inducing summer dormancy, and growth can persist under long days and high temperatures as far as water is available. Notably, once it enters into a dormant state, further irrigation will not cause growth or any significant physiological activities, under long days and high temperatures [4]. Similarly, our attempts to resume growth by irrigating *Z. dumosum* shrubs in the middle of August (2009) failed. In contrast, Terwilligner and Zeroni (1994) reported that irrigation of *Z. dumosum* in the middle of July resulted in the emergence of new leaflets (that is, new compound leaves emerging from the axil of the remaining phyllode/petiole) within two weeks. These controversial results may be attributed to the site of study. Accordingly, the Terwilligner and Zeroni (1994) study was conducted on a northeastern-facing slope, which is expected to dry out slowly relative to the southeastern-facing slope (the site of the present study). We, therefore, assume that the appearance of new leaves following irrigation in the middle of July is because the shrub is not yet fully dormant and can respond to ephemeral inputs of water by resuming growth.

The winter that represents the growing season poses difficulties to desert plants because the temperature largely fluctuates. Rainfall is low and is often highly variable both in space and time. Consequently, desert plants have inherent capabilities to withstand extreme seasonal climate changes and fluctuations in resource availability via a multitude of strategies (morphological and molecular) that operate together to bring about plant survival in the desert ecosystem. We described in this paper some molecular mechanisms operating in the desert plant *Z. dumosum* under seasonal climate change. Seasonal growth activity during the wet season is highly correlated with the rainfall and associated with the transcriptional epigenetic marker of histone H3 methylated at lysine 4 [36,37]. Accordingly, in years with a high amount of rainfall and a balanced rainfall distribution, the permissive epigenetic mark (H3K4me2) persists for a long period ranging from January to June or July [3]. However, in years with poor and unbalanced distribution of rainfall, H3K4me2 is relatively low and restricted to months experiencing a generous amount of rainfall. The disappearance of H3K4me2 from petioles during the summer suggests complete withdrawal from growth and entry into summer dormancy as soon as the dry season starts. Therefore, the epigenetic marker H3K4me2 appears to be a reliable indicator for plant growth activity in a variable desert environment.

In Arabidopsis, DNA methylation in the context of CHG appears to be coupled to histone H3K9 methylation in a feedback loop, which involves two epigenetic factors known as CMT3 and SUVH4/KYP. Accordingly, SUVH4/KYP methylates histone H3K9 generates a binding site for CMT3 that methylates cytosine in the context of CHG [10,11]. Consequently, methylated CHG sites serve as binding sites for SET and Ring finger Associated (SRA)/YDG domains-containing proteins such as SUVH4/KYP, which generates a feedback loop that expands both DNA and H3K9 methylation. This leads to chromatin compaction and gene silencing [12]. Loss of CHG methylation found in the *suvh4/kyp* mutant mimicked the loss of CHG methylation observed in the *cmt3* mutant [38]. Therefore, histone H3K9 methylation is required for CHG methylation mediated by CMT3. The findings that *Z. dumosum* does not possess di-methyl and tri-methyl H3K9 [3] prompted us to investigate the consequences for DNA methylation. The data presented in this paper clearly showed that DNA methylation is not affected in *Z. dumosum* in spite of a lack of di-methyl and tri-methyl H3K9. This can be explained by the persistence in *Z. dumosum* of monomethyl H3K9, which might be sufficient for directing CMT3 non-CG methylation genome-wide. Indeed, CMT3 can bind efficiently to H3K9 when it is mono-methylated, di-methylated, or tri-methylated [9]. Lastly, we observed changes in DNA methylation during the transition from the wet to the dry season and the acquisition of dormant state linking *Z. dumosum* seasonal growth with epigenetic reprogramming of gene expression. Similarly, changes in the DNA methylation pattern were observed during winter dormancy in an apple [39]. The effect of stress on epigenetic modification of histones and DNA has been well documented and is assumed to contribute to plant stress tolerance [40,41,42].

Proteome data of acid-soluble proteins extracted from petioles during the wet and the dry seasons revealed some molecular means for acquiring a dormant state as well as maintaining cellular integrity. Accordingly, the transition into the dry season resulted in the disappearance or significant reduction in ribosomal proteins. This massive reduction in ribosomal proteins might halt protein synthesis and facilitate the cessation of growth and entry into a dormant state. The proteome data also pointed to a reduction in photosynthesis and respiration activities as evidenced by reduction in hydrolytic enzymes residing in the chloroplasts such as Fructose-1,6-bisphosphatase 1, Ferredoxin-dependent glutamate synthase, and ATP synthase subunit delta as well as in the mitochondria such as Dihydrolipoyl dehydrogenase and NADH-ubiquinone oxidoreductase (Table 1). It appears that, during the dry season, certain vital activities are either lost or significantly reduced in the remaining petioles including protein synthesis, photosynthesis, and respiration, which allow dormancy and survival under extreme environments. This dormant state is further reinforced by increasing the levels of the polyubiquitin precursor protein and ubiquitin conjugated to ribosomal proteins known as orthologs of ubiquitin-RPL40 and ubiquitin-RPS27. The covalent conjugation of ubiquitin to substrate proteins (via ubiquitin lysine 48) directs them to undergo proteolysis in the proteasome system [43], which might be instrumental in controlling the dormant state attained by cells during the dry season. The function of ubiquitin conjugated to ribosomal proteins is not fully understood. Recent work in animals highlighted the central roles of ubiquitin-coding gene UBA52, encoding for a mono-ubiquitin fuses at its C-terminus to the ribosomal protein L40 (RPL40), and in the regulation of the physiological level of ubiquitin and ribosomal functionality [44]. The accumulation of ubiquitin-RPL40 and ubiquitin-RPS27 in *Z. dumosum* petioles during the summer may be attributed to a lack of de-ubiquitinases, which are enzymes that recycle ubiquitin from ubiquitin conjugates or ubiquitin precursors [45].

We observed seasonal variation in HSP proteins associated with temperature variation, particularly, small HSP proteins found in high amounts during the dry season and in low amounts during the wet season (Figure 3). This is consistent with the proteome data showing an increase in the levels of HSP proteins, which include small HSP17.4, HSP90, and HSP70B found during the dry season. Expression of small HSP proteins (sHSPs) is induced following exposure to various stress conditions including heat, drought, and salt and has been correlated with stress tolerance [28]. HSP70B appears to be expressed exclusively during heat stress [30] and might be involved in thermo-tolerance. It assists in refolding of denatured proteins [46]. In the absence of stress, sHSPs expression is restricted to certain developmental stages including embryo and pollen development and germination [28]. The mechanism by which sHSPs confer stress tolerance is not well understood. Some sHSPs as well as HSP90 were shown to act as molecular chaperones stabilizing and assisting proper protein folding [47,48,49]. This function may explain why these non-basic HSP proteins are extractable in acidic buffer. Accordingly, we suggest that HSP proteins are physically associated with basic proteins during the dry season (and, therefore, are co-extracted with them) to maintain the integrity of cells by protecting proteins from unfolding and aggregation and keeping them in a competent state for correct folding once growth is resumed.

## 5. Conclusions

Multiple mechanisms evolved in the desert plant *Z. dumosum* to allow for its survival under extreme seasonal climate changes. Like other plants, its growth cycles and productivity are highly dependent on the availability of water [50,51] and are associated with the presence of the permissive epigenetic marker of di-methyl H3K4. Clearly, acquisition of a dormant state during the dry season is an important mechanism for adaptation and survival to multiple abiotic stresses, which are expected to increase in frequency and severity due to global warming [5]. Summer dormancy in *Z. dumosum* is achieved by depriving the permissive epigenetic marker di-methyl H3K4, which, in turn, facilitates the acquisition of compact chromatin conformation [3] as well as by a significant reduction in proteins involved in basic cell functions such as protein synthesis, photosynthesis, and respiration. Some proteins may be associated with HSP-chaperones whose levels are increased during the dry season to ensure the integrity of cellular components. Certain epigenetic markers such as di-methyl and tri-methyl H3K9 have been lost in *Z. dumosum* [3] and in all examined *Zygophyllaceae* species that inhabit dry and semidry regions of the world [7]. This suggests that lessening epigenetic constraints might have an adaptive value in variable, unpredictable environments, which provides plants with opportunistic capabilities such as a prompt response to their ever-changing environment to allow for rapid exploitation of transient input of resources. Although multiple mechanisms were evolved to bring about plant tolerance in variable desert environments, epigenetic means appear to be central in controlling gene expression and are, therefore, important for stress memory and adaptation in plants [14,52]. We, therefore, anticipate that plants in general—and desert plants in particular—may be resilient to climate change [53] based on the existence of a plethora of mechanisms for stress tolerance and the potential to attenuate the effect of extreme environmental conditions through prompt manipulation of the epigenetic landscapes.

## Figures and Tables

**Table 1:** List of nucleic acid binding proteins, oxireductases/dehydrogenases and hydrolases, which are significantly down-regulated in *Z. dumosum* petioles in the dry season. (M, mitochondria; Ch, chloroplast).

Nucleic Acid Binding Proteins			
Accession	Arabidopsis Gene ID	Gene Name/Gene Symbol Ortolog	Ratio Wet/Dry
Q93VC7	At5g30510	30S ribosomal protein S1; RPS1 **(Ch)**	30.40
F4J3P1	At1g04480	60S ribosomal protein L23; RPL23A	5.71
Q8VZB9	At1g08360	60S ribosomal protein L10a-1; RPL10AA	12.02
O04527	At1g70190	F20P5.9 protein	Not found in dry season
Q9SJ36	At2g05220	40S ribosomal protein S17-2; RPS17B	Not found in dry season
P46286	At2g18020	60S ribosomal protein L8-1; RPL8A	Not found in dry season
Q39244	At2g47580	U1 small nuclear ribonucleoprotein A; U1A	64.76
A0A1P8AW31	At3g02540	Ubiquitin receptor RAD23c; RAD23C	Not found in dry season
Q9LK61	At3g13120	30S ribosomal protein S10; RPS10 **(Ch)**	Not found in dry season
O23290	At3g23390	60S ribosomal protein L36a; RPL36AA	Not found in dry season
P42036	At3g52580	40S ribosomal protein S14-3; RPS14C	5.63
P38666	At3g53020	60S ribosomal protein L24-2; RPL24B	7.56
Q9M352	At3g53740	60S ribosomal protein L36-2; RPL36B	59.70
A8MS83	At3g55280	60S ribosomal protein L23a-2; RPL23AB	21.34
Q9LZH9	At3g62870	60S ribosomal protein L7a-2; RPL7AB	Not found in dry season
Q9M1X0	At3g63190	Ribosome-recycling factor; RRF **(Ch)**	23.60
P49693	At4g02230	60S ribosomal protein L19-3; RPL19C	34.07
Q9SZD6	At4g29060	Elongation factor Ts, emb2726 **(M)**	Not found in dry season
Q9M0E2	At4g29410	60S ribosomal protein L28-2; RPL28C	4.44
Q93VG5	At5g20290	40S ribosomal protein S8-1; RPS8A	14.12
Q9FI15	At5g44500	Small nuclear ribonucleoprotein-associated protein	Not found in dry season
P55228	At5g48300	Glucose-1-phosphate adenylyltransferase small subunit	Not found in dry season
A8MQA1	At3g49010	60S ribosomal protein L13-1; RPL13B	6.94
Q9FNP8	At5g61170	40S ribosomal protein S19-3; RPS19C	Not found in dry season
**Oxireductases/dehydrogenases**			
Q9M5K3	At1g48030	Dihydrolipoyl dehydrogenase 1, **(M)**	8.71
F4HNZ6	At1g12900	Glyceraldehyde-3-phosphate dehydrogenase, **(Ch)**	4.6
Q9ZP06	At1g53240	Malate dehydrogenase 1, **(M)**	4.45
A0A1P8BD41	At5g52840	NADH dehydrogenase 1 alpha subcomplex subunit 5, **(M)** Complex I, non-core accessory subunit B13	Not found in dry season
F4JWS9	At5g25450	Cytochrome b-c1 complex subunit 7-2;QCR7-2 Complex III	3.99
Q9ZNZ7	At5g04140	Ferredoxin-dependent glutamate synthase 1, **(Ch/M)**	Not found in dry season
Q94B78	At4g33010	Glycine dehydrogenase (decarboxylating) 1, GLDP1 **(M)**	Not found in dry season
A0A1P8B993	At3g14420	Peroxisomal (S)-2-hydroxy-acid oxidase GLO1	Not found in dry season
**Hydrolases**			
Q9SSS9	At4g09650	ATP synthase subunit delta **(Ch) AtpD;** HCP	not found in dry season
P0DKC4	At5g36790	Phosphoglycolate phosphatase 1B, PGLP1B **(Ch)**	not found in dry season
P25851	At3g54050	Fructose-1,6-bisphosphatase 1, CFBP1 **(Ch)**	not found in dry season
Q9ZNZ7	At5g04140	Ferredoxin-dependent glutamate synthase 1, **(Ch/M)**	not found in dry season
O80860	At2g30950	ATP-dependent zinc metalloprotease FTSH 2 **(Ch)**	5.7

**Table 2:** List of proteins significantly up-regulated in *Z. dumosum* petioles in the dry season. Ch, chloroplasts.

Accession	Related to Arabidopsis Gene ID	Gene Name/Gene Symbol Ortolog	Ratio Wet/Dry
Q9ZUC1	At1g23740	NADPH-dependent alkenal/one oxidoreductase **(Ch)**	0.1
O80977	At2g14740	Vacuolar-sorting receptor 3	0.1
OAP01990.1	At3g40120	HSP17.4	0.2
Q9SRZ6	At1g65930	Cytosolic isocitrate dehydrogenase [NADP]	0.2
P27323	At5g52640	Heat shock protein 90-1	0.2
Q9SYT0	At1g35720	Annexin D1	0.3
Q94JQ4	At3g20390	Reactive Intermediate Deaminase A **(Ch)**	0.3
O49006	At3g14310	Pectinesterase/pectinesterase inhibitor 3	0.3
A0A1I9LT03	At3g03250	UDP-GLUCOSE PYROPHOSPHORYLASE 1	0.3
ABH08753.1	At4g05320	ubiquitin	0.4
Q42202	At2g36170	Ubiquitin-60S ribosomal protein L40-2	0.4
F4IGK5	At2g21250	NAD(P)-linked oxidoreductase superfamily protein	0.4
O03986	At5g56000	Heat shock protein 90-4	0.5
P59232	At2g47110	Ubiquitin-40S ribosomal protein S27a-2	0.5
F4JZ46	At5g66190	Ferredoxin-NADP reductase	0.5
Q9S9N1	At1g16030	Heat shock 70 kDa protein 5	Not found in wet season
Q9ZSJ7	At3g24160	Peroxisome membrane protein (PMP)	Not found in wet season

## Supplementary Materials

The following are available online at http://www.mdpi.com/2223-7747/7/3/59/s1. Table S1: A Combined list of proteins recovered from Zygophyllum petioles collected in the field during the wet (April) and the dry (October) seasons.
